# The association of diabetes literacy with self-management among older people with type 2 diabetes mellitus: a cross-sectional study

**DOI:** 10.1186/s12912-019-0354-y

**Published:** 2019-08-16

**Authors:** Utami Rachmawati, Junaiti Sahar, Dwi Nurviyandari Kusuma Wati

**Affiliations:** Faculty of Nursing Universitas Indonesia, Jalan. Prof. Dr. Bahder Djohan, Depok, West Java 16424 Indonesia

**Keywords:** Diabetes literacy, Diabetes self-management, Older people

## Abstract

**Background:**

Diabetes has become one of public health problem up until now. As the disease progressed, it might lead to increasing complication as well as death related to them. Diabetes as chronic disease in older people can lead to more vulnerable conditions if they fail to carry out a proper diabetes self-management. Diabetes literacy is an internal factor affecting how the older people go about their diabetes management routines. This study aimed to describe diabetes literacy of the older people and identify the relation of diabetes literacy to diabetes self-management of the older people with T2DM in selected areas of Depok City, West Java, Indonesia.

**Method:**

A cross-sectional observational study was utilized and used 106 samples of older people individuals with T2DM, all of whom were chosen via cluster sampling. This research took place in five selected areas under the supervision of three public health center in Depok City. The data were analyzed using a bivariate t-independent test, the Pearson product-moment correlation, and logistic regression for multivariate analysis to determine the relationship of independent and dependent variable.

**Result:**

This research shows a significant correlation between diabetes literacy and diabetes self-management (*p* = 0,011).

**Conclusion:**

Diabetes self management is associated with diabetes literacy in older people with type 2 diabetes mellitus. Diabetes literacy should be considered when assessing and addressing diabetes-specific health education needs.

## Background

Degenerative health problems that the older people experience are closely related to the aging process as well as biological and lifestyle risk factors. One of the health problems that often occurs in old age due to the various factors is Type 2 Diabetes Mellitus (T2DM). There are 387 million people all over the world with T2DM and this number continues to grow [1]. The World Health Organization (WHO) report (2015) showed that diabetes is one of the main causes of high mortality worldwide. The International Diabetes Federation records in 2015 also indicated that the prevalence of T2DM in Indonesia increases with age and reaches its peak at the age of 60–64 of nearly 15%. T2DM ranks fifth among the contributing diseased in old age [2]. The figure rose every year from 1.1% in 2007 to 2.1% in 2013, and Depok as one of the cities in West Java also shows a significant increase in the number of diabetics from as many as 4,834 in 2015 to 7,365 in 2016.

Diabetes mellitus is characterized by the increased levels of glucose in the blood or hyperglycemia due to abnormalities in insulin secretion or insulin action, or both [3]. This can be seen from the reports of a research conducted in Cimanggis Sub-municipality which indicate that there are still 45% of older people (out of a total of 99 respondents) with inadequate knowledge of diabetes, 25% of whom do not know about diabetes and its complications. From the results of the preliminary research conducted by the researcher in 2016, it is found that compliance to diabetic self-management is not yet optimal, indicated by a number of 60% respondents still consuming restricted food such as high calories or sugar level and 26.7% not exercising regularly. This shows that the increasing number of older people T2DM indicates non-optimized knowledge and implementation of diabetes self-management.

The results of regular health monitoring by Posbindu (the community healthcare center for older people people) indicate that there was lack of older people participation. This finding was shown in the Posbindu staff’s report stating that the older people who come are always the same people every month. There are still older people who have diabetes who do not come regularly for a check-up to Posbindu [4]. This is not only the case in one village, but also in other areas as shown in a research by Rusdianingseh in 2014 reporting that there is still lack of enthusiasm in the community causing Posbindu to not have a complete record of clients with T2DM in that region. The Health Program for Older people with Diabetes Mellitus (LANSET DM in Bahasa) developed by Ratnawati in 2013 has not been duplicated in other villages. Interventions related to the issue of older people diabetes have also been implemented through health education and a direct intervention through home visits [5]. Nevertheless, the secondary data from the local Community Health Center in 2016 still indicate the persisting high rates of T2DM in the older people.

Health education is one of the multidisciplinary approaches to overcome the problem of older people with T2DM and relies on the cognitive ability and health literacy [6, 7]. Health literacy refers to the ability of person to seek, process, understand, and apply the necessary information regarding their health [8]. The diabetes literacy components that can be observed from this study include Basic/Functional Literacy, Communicative/ Interactive Literacy, and Critical Literacy [9]. Health literacy is known to determine the successful achievement of health outcomes [10], as well as improve patients’ diabetes self-management [11]. Studies suggest that low health literacy lead to poor self-management knowledge and abilities [12], poorer level of glycaemic control [13] and higher level of HbA1c in people with diabetes [14].

Low health literacy can also indicate that the health promotion techniques are not used appropriately [8]. Low health literacy is linked to the declining health status of the older people and results in low compliance to disease prevention programs [15]. A health literacy study on T2DM patients in Taiwan shows that health literacy has an indirect impact on the patients’ blood sugar control [16]. The study also recommends improving diabetes self-care through the betterment of health literacy and self-efficacy of patients [17]. As such, health literacy is closely related to a patient’s health behavior.

Promotional actions are necessary to minimize burdens for the individuals, families, and governments impacted by diabetes. Promotional programs can reduce the costs of inpatient hospital care, analogue insulin, and outpatient care [18]. The above explanation describes the factors in the older people that can affect the diabetes self-management in their treatment of diabetes. Diabetes literacy is a factor related to diabetes self-management of the older people with T2DM, there are already some studies about this however, there is a dearth information regarding this matter espescially in Indonesia. This study aimed to describe diabetes literacy of the older people in Depok City and identify the relation of diabetes literacy to diabetes self-management of the older people with diabetes.

## Methods

### Study area and study period

The study was carried out in several areas in Depok City from February to June, 2016. There were 3 Puskesmas (the Regional Public Service Agency of Community Health Service) used in this study namely, Puskesmas Tugu, Puskesmas Cimanggis, and Puskesmas Sukatani. On each Puskesmas chosen, there were two selected municipality for this study, except Puskesmas Tugu with only one area selected.

### Study design

This research used the descriptive correlational research design with the cross-sectional observational approach.

### Source of population

All of the older people with T2DM in Depok City.

### Study population

All the sampled older people with T2DM who have been living in area under the supervision of Puskesmas Tugu, Puskesmas Sukatani, and Puskesmas Cimanggis of Depok City, West Java, Indonesia.

### Selection criteria

#### Inclusion criteria

Sampled older people who diagnosed with T2DM, able to communicate, read and write in Bahasa.

#### Exclusion criteria

Older people with T2DM who were having difficulties in both speaking and hearing.

### Sampling

#### Sampling size determination

Samples were collected in 5 different areas according to the sample’s inclusion criteria. The sample size was determined using the following assumption and a single population (p) n = $$ \frac{Z\raisebox{1ex}{$1$}\!\left/ \!\raisebox{-1ex}{$2$}\right.{\alpha}^2 PQ}{d^2} $$ was employed. Where n is sample size desired, z^2^ is a standard normal score of 95% of confidence interval = 1.96, d is degree of accuracy = 0.05 and *p* = 50%, which was the estimated population proportion of the older people with diabetes in five selected areas. Since the total source of the population was less than ten thousand which was 96; then by adding 10% for the possible non-response rate, a total sample size of 106 was obtained. Proportional allocation was used to allocate the sample in five selected areas.

#### Sampling procedure

Sampling was conducted using purposive sampling in which areas were previously used to be the work-study area of nursing chosen. The cluster sampling method were used as sampel was taken from different areas. After taking proportion in each area, 106 older people with T2DM where chosed randomnly from the data taken from both public health center and social worker datas.

### Data collection procedure

Data were collected using a structured-self administered questionnaire that were distributed and collected by enumerators from 15 to 31 June 2017.

### Data collection tool

The three instruments used in this research included the socio-demographic questionnaire —which queried age, sex, educational background, household income, ethnicity, family history of diabetes, and information media used for daily and health education needs—, the diabetes literacy questionnaire, which was a diabetes literacy questionnaire adapted from Functional, Communicative, and Critical Health Literacy Scale [19], and the diabetes self-management questionnaire used by Masi in 2016, which is adopted and translated into Bahasa from The Diabetes Self-Management Questionnaire (DSMQ) [20]. The validity test results show that the correlation of the items questionnaire from diabetes literacy ranging from 0,429–0,742 and for the DSMQ ranging from 0,376-0,797. However, there is 1 item from diabetes literacy questionnaire that was not valid (0,197). The sentence from this item was modified so that it can be used. The reliability test results show that the value of Cronbach’s alpha for the diabetes literacy questionnaire is 0.743, and for the diabetes self-management questionnaire 0.667, and based on those values they are declared to have adequate internal consistency as a research questionnaire.

### Data quality assurance

**Fig. 1:**
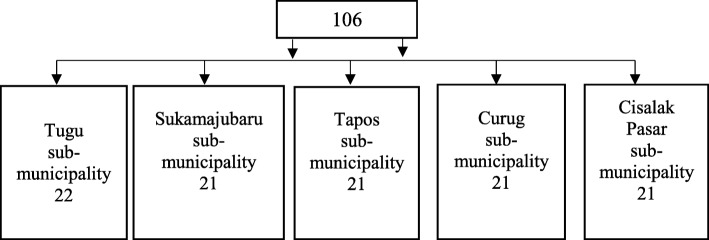
Schematic sampling procedure (*n* = 106)

To assure data quality, orientation was given to experienced enumerators. Enumerators as data collectors responsible for checking the missing answers at each points. The data were also checked during entry and before analysis. Tje schematic sampling procedure is described in Fig. 1.

### Study variable

*Dependent variable*

### Diabetes self-management

*Independent variable*

### Diabetes literacy

#### Data analysis procedure

The data were entered and analyzed using statistical data analysis software SPSS version 17. The statistical analysis was made at the 95% confidence level. The bivariate analysis to determine the significance of the relationship between the diabetes self-management variable and the diabetes literacy variable was conducted using Pearson product-moment correlation test. The multivariate analysis was conducted using logistic regression.

#### Ethical consideration

There was no physical and psychological harm done to the participant. This research ensures that no force was applied to praticipants in joining the study. The study was also approved by the Faculty of Nursing University of Indonesia Reaseach Ethic Committee (reference: No.100/UN2.F12.D/HKP.02.04/2017).

## Result

### Socio demographic characteristics of the respondents

In this study, 106 older people with diabetes were recruited. Among the participants, median age and income of the respondents are 64 years (95% CI: 64.51–66.25), and 2,000,000 IDR (95% CI: 2,165,566.04–1,937,586.77). The detailed characteristics of the respondents are described in Table 1.

**Table 1:** Socio demographic Characteristics of Respondents (n = 106)

Characteristic	Frequency	%
Sex		
Male	34	32.1
Female	72	67.9
Ethnicity		
Betawinese	33	31.1
Javanese	57	53.8
Others	16	15.1
Educational level		
Elementary Education	52	38.1
Secondary Education	45	42.5
Higher Education	9	8.5
Family History of Diabetes		
No history	52	49.1
With history	54	50.9
Information Media Used^a^		
Magazines	3	2,8
Books	6	5,7
Radio	31	29,2
Television	98	92,5
Internet	14	13,2
Health Information From The Past Month^a^		
Printed media (newspaper, magazines, books)	10	9,4
Brochures from clinics or other medical settings	30	28,3
Posters from clinics or other medical settings	14	13,2
Radio	13	12,3
TV	38	35,8
Internet	12	11,3
Family and friend	64	60,4
Health care providers at Public Health Center	54	50,9
Health care providers at other medical settings	49	46,2

^**a**^respondents may choose more than one option for their answer

The characteristics of the respondents show that their average age is 65 years, the majority of them are of the Javanese and Betawi ethnic groups, there is a balanced proportion of low and middle education, their average income is 2 million IDR, and most have a family history of diabetes. Respondent may choose more than one option regarding information media used and the majority of them use televisin. The big number of health information were gained from family and friend. Furthermore, the mean of diabetes literacy in respondents was 42,95 (95% CI: 41,55 - 44,36). Diabetes literacy and diabetes self-management are described in Table 2.

**Table 2:** Association of Diabetes Literacy to Diabetes Self-Management (n = 106)

Variable	Diabetes Self-Management	
	r	p *value*
Diabetes Literacy	0.246	0.011*

*signficant when p *value <* 0.05

Diabetes literacy significantly affects diabetes self-management with the *p* value of 0.011. However, the coefficient correlation 0,246 shows weak relationship of these variables. This shows that the better someone’s diabetes literacy, the better their diabetes self-management. Respondents with good diabetes literacy will have the ability to search for, process, and apply good health information. Thus, if the information needed to implement diabetes self-management has been well received and understood, diabetes self-management will also be well implemented. Moreover, the analysis of diabetes literacy components are described in Table 3.

**Table 3:** Components Analysis of Diabetes Literacy (n = 106)

Diabetes Literacy	Lower		Higher	
	n	%	n	%
Basic/Functional Literacy	32	30,2	74	69,8
Communicative/Interactive Literacy	51	48,1	55	51,9
Critical Literacy	73	68,9	33	31,1

The final modeling from the final multivariate analysis after going through the candidate, interaction, and confounder testing process is described in Table 4. The result shows that the respondents with good diabetes literacy are two times more likely to perform good diabetes self-management compared to the patients with poor diabetes literacy after being controlled by age and income. This means that good diabetes literacy will be associated with good diabetes-related knowledge as well. Good knowledge of diabetes will be able to change one’s behavior, in this case in improving their diabetes self-management.

**Table 4:** Final Model Resulting from the Multivariate Analysis Related to Diabetes Self-Management (n = 106)

Variable	Beta	p *value*	Exp(B)	OR (CI 95%)
Age	0.983	0.025	2,673	0.440
Income	0.795	0.066	2,214	0.432
Diabetes literacy	0.906	0.038	2,476	0.437
*constant*	−3.794	0.022	0,022	1.221

## Discussion

The results of the statistical tests for this research indicate that there is a significant relationship between diabetes literacy with diabetes self-management. These results are supported by a research conducted by Bailey, et al. in 2014 which suggests that low health literacy is associated with low knowledge of diabetes [21]. Another similar study also explains that the higher the health literacy the higher the participation of one’s self-management [12]. Low health literacy is synonymous with a declining health status of the older people and results in low compliance to disease prevention programs [15]. Ishikawa and Yano also state that diabetes patients with a good level of health literacy will tend to demonstrate a good level of participation and diabetes self-care efficacy [22].

Another study that supports these results was conducted by Fransen, et.al who states that low health literacy is associated with low knowledge of diabetes and leads to poor self-management as well [23]. A health literacy study on type-2 diabetes patients in Taiwan shows that health literacy has an indirect impact on the patients’ blood sugar control as one of the components of diabetes self-management of patients [16]. This finding, however, contradicts that of Al-Sayah, Majumdar, and Johnson (2015), who state that health literacy does not have a direct impact on the health status of diabetics [13].

The component of functional literacy of the respondents has varying assessments. However, there are more respondents who state that the counseling or health information that they receive today is delivered with ease, that they now rarely find difficult terms or writings that are too small, and that the information is easy to understand. Respondents who need help to understand health information amount to one-third of the total respondents. White reveals that health literacy is related to one’s experience about the structure of health information such as brochures for patients [24]. This shows that the role of media is quite important in delivering health information to respondents.

Older people with declining physical functions will certainly experience a setback in the aspects of eyesight and cognitive ability. Therefore, it is not uncommon to find older people who express having difficulties or need help when receiving information. As many as a third of the respondents in this study still need help from someone else in reading or understanding a piece of health information. The respondents consider that their family should also know and read the information delivered to them to help them remember it more easily. This statement from the respondents is consistent with a study by Strizich, et al. in 2016 which states that older people with declining cognitive abilities and minimal family support will be more likely to suffer from uncontrolled diabetes [25].

During the data collecting, a spontaneous interview showed that communicative literacy ability of the respondents is fairly good because the respondents have begun to seek and gather information related to their health. This may be due to the research taking place in the practice area for nursing students, which is also a factor enabling the high rate of search and application of health information in this research. Even though the use of media information is not statistically affecting self-management, it might be taken into consideration that it played as contributing factors. The respondents of this research have often received information from the work-study nursing students as well as from the health workers who are regularly on duty in Posbindu (the community healthcare center for older people) and community healthcare center. Then, since the respondents received the information they have made attempts to understand and apply it in their daily life.

The research conducted by von Wagner, Wolf, Steptoe, and Wardle in 2009 shows that self-management requires certain abilities, such as the ability to understand information and how lifestyle can affect diabetes [26]. The ability to seek and collect health information is also affected by the existing access to health information. Pawlak finds that information technology is one of the determinants of health literacy [27]. Ease of information collection will certainly increase the search for and collection of information by respondents even further. The research conducted by Santosa showed that one factor that has an impact on health literacy is access to health information [28].

The ability to seek and collect health information is also affected by the access to health information available. Ferawati, Hasibuan, and Wicaksono state that diabetes management is also affected by the factor of information support [29]. The results of this study are supported by the results of a similar research related to access to health information and diabetes care management conducted by Lai et.al, wherein they state that decisions on health made and implemented by individuals are affected by comprehensive, accessible health information that is appropriate based on their needs and socio-cultural backgrounds [30]. This means that the individual’s diabetes self-management will be good if the necessary health information is easy to obtain and meets the individual’s needs as well as characteristics such as education and cultural values adopted by the individual concerned.

Informational support from the family is known to also impact diabetes self-management. The results of this research show that half of the respondents get health information from their friends and family. This finding is consistent with that of Netismar in 2017 who states that informational support from the family is one of the motivations for the implementation of diabetes self-management [31]. The majority of research respondents have received health-related information during the past month. Apart from their family, health information that they received also came from the health workers at public health center, Posbindu, and elsewhere. This shows that access to individual health information is well functioning and there is also the role of health personnel as one of the health information providers during the diabetes treatment undergone by the respondents, and these affect the communicative literacy aspect.

These findings are reinforced by a research conducted by Santosa wherein she states that health information provided by health personnel can now be more easily digested, only that it is not given frequently enough [28]. For example, a respondent stated that during the doctor visit, a brie counselling was given with some adjustments in the way that it’s easier for patient to understand medical terms. The respondents do not visit the health care facility every day, so there is a need for other media where health information can be easily accessed. Interviews with the respondents indicate that television is more interesting because they can see for themselves what is being delivered. This is in line with the findings of Newblod and Campos (2011) which assert that radio and television are more effective in terms of audience reachability and repeatability of the news compared to print media [32]. The explanation shows that the role of print media is not very efficient in delivering health education to the older people.

The gap filled by this research related to diabetes literacy and diabetes self-management is found in the minimal amount of research that examines diabetes literacy in more specific terms and its relationship with diabetes self-management. The findings of this research indicate that diabetes self-management will be good if accompanied by efforts to obtain, process, and apply good health information as well. Educational background and functional status will also represent different levels of diabetes literacy in the older people and, as such, they render the health education efforts undertaken ineffective. The results of this research also show that the older people access health information mostly on television as an audiovisual device. Therefore, nurses ought to have the ability to design health education using audiovisual media that must take into consideration the aspects of age and education of the older people.

## Conclusion

There is a significant relationship between diabetes literacy with diabetes self-management of the older people with diabetes, in that the better their diabetes literacy the better their self-management. Diabetes literacy in the older people can improve their diabetes self-management alongwith ability to seek and apply information on diabetes available through the use of suitable media information. It is expected that the results of this research will able to provide an input and materials for consideration in order to overcome the problems of older people diabetes by considering the aspect of diabetes literacy when assessing and addressing diabetes-specific health education needs, one method being individual health education interventions conducted using audiovisual media.

## Abbreviations

CI: Confidence interval
DRPM: Directorate of research and community engagement (Direktorat Riset dan Pengabdian Masyarakat in Bahasa)
DSMQ: The diabetes self-management questionnaire
Exp(B): Exponent Beta
LANSET DM: A term used in Bahasa for the health program for older people with diabetes Mellitus
PITTA: Internationally Indexed Publication for Final Assignment (Publikasi terindeks Internasional untuk Tugas Akhir in Bahasa)
SD: Standard of deviation
T2DM: Type 2 diabetes mellitus
